# Low-dosage metronomic chemotherapy and angiogenesis: topoisomerase inhibitors irinotecan and mitoxantrone stimulate VEGF-A-mediated angiogenesis

**DOI:** 10.1111/j.1600-0463.2011.02830.x

**Published:** 2012-02

**Authors:** PER ALBERTSSON, BO LENNERNäS, KLAS NORRBY

**Affiliations:** 1 Departments of Oncology, Sahlgrenska Academy, University of GothenburgGothenburg, Sweden; 2 Departments of Pathology, Sahlgrenska Academy, University of GothenburgGothenburg, Sweden

**Keywords:** Angiogenesis, VEGF, metronomic chemotherapy, irinotecan, mitoxantrone, rat, reactive oxygen species

## Abstract

Albertsson P, Lennernäs B, Norrby K. Low-dosage metronomic chemotherapy and angiogenesis: topoisomerase inhibitors irinotecan and mitoxantrone stimulate VEGF-A-mediated angiogenesis. APMIS 2011.

Metronomic chemotherapy with cytotoxic agents has been shown to inhibit angiogenesis and, consequently, tumor growth by targeting vascular endothelial cells (ECs). In these regimens, anti-tumor activities additional to anti-angiogenesis may operate. Moreover, chemotherapy typically generates reactive oxygen species in targeted ECs, which can affect angiogenesis. The aim of the present study was to assess the systemic effect of low-dosage metronomic treatment with either irinotecan or mitoxantrone on angiogenesis induced by VEGF-A. Angiogenesis was induced in normal adult rat mesentery by intraperitoneal injection of a low dosage of VEGF-A. Thereafter, irinotecan and mitoxantrone were infused separately continuously at minimally toxic dosages for 14 consecutive days via a subcutaneous osmotic minipump. Angiogenesis was assessed in terms of objective and quantitative variables using morphologic and computerized image analyses. Irinotecan or mitoxantrone significantly stimulated angiogenesis, with ironotecan increasing angiogenesis by 104%, when compared with the vehicle-treated animals. Low-dosage metronomic chemotherapy with irinotecan or mitoxantrone stimulates angiogenesis in the normal mesentery of rats, probably by inducing low-level oxidative stress in the targeted ECs. Whether or not this pertains to tumor angiogenesis may be difficult to confirm, as several anti-tumor modes may operate during low-dosage metronomic chemotherapy.

In 2000, Browder and colleagues (1) reported that frequent systemic administration or continuous infusion with cyclophosphamide at a minimally toxic dose inhibited basic fibroblast growth factor (bFGF)-mediated angiogenesis in the mouse corneal micropocket assay, induced the apoptosis of vascular endothelial cells (ECs) in tumor microvessels, and suppressed the growth of transplantable mouse tumors. In the same year, Klement and coworkers (2) reported on the following effects of frequent intraperitoneal (i.p.) injections of low dosages of vinblastine in mice: (i) no significant effect on body-weight; (ii) significantly reduced vascularity of subcutaneous (s.c.) Matrigel pellets that contained bFGF; (iii) significant albeit transient xenograft tumor regression; (iv) diminished tumor vascularity; and (v) direct inhibition of tumor angiogenesis in SCID mice. The presumptive target cell in these studies was the normal angiogenically activated EC, and continuous chemotherapy-induced apoptosis of ECs in the vascular bed of tumors has been shown to precede the apoptosis of nearby neoplastic cells (3). As tumor progression is angiogenesis-dependent, the benefit of this continuous scheduling is that it is significantly less toxic and has more potent anti-tumor effects than conventional, spaced-out, high-dosage chemotherapy, owing in large part to the fact that extended periods of treatment become feasible (4). An additional advantage of continuous chemotherapy is that the targeted ECs are rapidly exposed to the circulating drugs, and as ECs are considerably more stable genetically than neoplastic cells, they are less prone to mutate and acquire drug resistance. In addition to its effects on ECs and pericytes, metronomic chemotherapy affects neoplastic tumor cells, tumor stroma cells (including fibroblasts, macrophages, and mast cells), platelets, lymphocytes, as well as precursor ECs in the bone marrow, which may significantly influence tumor angiogenesis and growth.

The success of continuous chemotherapy scheduling in tumor-bearing mice, in which the tumor cells were cyclophosphamide-resistant, led to this treatment being labeled anti-angiogenic chemotherapy (1). Continuous treatment scheduling is now more generally referred to as metronomic chemotherapy. Metronomic chemotherapy has been used in a multitude of pre-clinical studies (5–8), as well as in clinical trials (9–15). In many of these studies, the combination of metronomic chemotherapy and conventional anti-angiogenic agents was tested, which often enhanced the anti-tumor effect while making it difficult to evaluate the efficacy of the purported anti-angiogenic effect *per se* in the tumors of continuous chemotherapy.

Recently, the mechanism by which cytotoxic chemotherapy affects the tumor vasculature was thought to include selective killing of ECs, suppression of circulating EC precursor cells and/or increasing levels of endogenous angiogenesis inhibitors, and decreasing levels of angiogenesis stimulators (16). In 2006, using the rat mesentery angiogenesis model, which differs in many important aspects from the corneal micropocket and the s.c. Matrigel pellet assays used by Browder et al. (1) and Klement et al. (2), respectively, as discussed elsewhere (17, 18), we reported that continuous low-dosage infusions of chemotherapeutics influenced VEGF-A (VEGF)-mediated angiogenesis in a drug-specific manner (19). In this model, cyclophosphamide [confirming the data of Browder et al. (1)], as well as vinblasine [confirming the data of Klement et al. (2)], and paclitaxel significantly suppressed angiogenesis, whereas cisplatin and 5-fluorouracil, paradoxically, significantly stimulated angiogenesis (19). Notably, neither doxorubicin nor epirubicin displayed significant effects (19, 20).

In the present investigation, we assessed the effect of continuous s.c. infusion of either irinotecan or mitoxantrone on angiogenesis induced by VEGF, which is a major pro-angiogenic factor in most experimental and human tumors. The tumor-free rat mesentery angiogenesis assay was used, and angiogenesis was measured in terms of unbiased, objective and quantitative variables. Notably, treatment with either of these drugs significantly augmented angiogenesis.

## Materials and Methods

### Animals

Adult young outbred male Sprague-Dawley rats (B&K Universal, Sollentuna, Sweden) were acclimatized to a standardized environment for at least 7 days, fed *ad libitum* and randomly allocated to weight-matched groups with two animals housed per cage (21). At the start of the pro-angiogenic i.p. treatment with VEGF, the mean body weights were 232.1 g and 179.3 g in the irinotecan and mitoxantrone experiments, respectively. Body weight was monitored daily. Given the rapid physiologic growth of adult rats (gaining approximately 60 g per week) drug-related, weight-gain retardation is a sensitive surrogate marker of toxicity, systemic well-being, anorexia, and failure to thrive. The Animal Ethics Committee of the University of Gothenburg approved this study. The ethical guidelines were those established by Workman et al. (22).

### Angiogenesis induction and a note on the mesentery assay

Rat rVEGF_164_ (564-RV/CF; R&D Systems Europe Ltd., Oxon, UK), which is the predominant VEGF isoform in rats, was diluted to 96 pmole/mL in the endotoxin-free saline used for infusion into patients, frozen and thawed and a volume of 5 mL was injected i.p. into the rats (23). This low-dosage treatment, given twice daily for 4.5 days, i.e., from Monday morning (Day 0) to Friday morning (Day 4) induces a robust sprouting angiogenesis response in the mesenteric test tissue, peaking at around Day 21 (23). Subsequent to the i.p. VEGF treatment, s.c. infusion with irinotecan or mitoxantrone was administered for 14 consecutive days. The VEGF treatment did not affect body-weight-gain, as compared with controls treated i.p. with the saline vehicle.

In similarity to most normal adult tissues, the membranous, small-gut mesentery in rats is natively vascularized (albeit sparsely), and significant physiologic angiogenesis is lacking in adult Sprague-Dawley rats (17). The test tissue is untouched mechanically until the experiment is concluded. The inflammatory stimulus of the test tissue is minimal, which ensures a high level of sensitivity, as inflammation induces angiogenesis. The assay replicates the clinical circumstances, as the test drugs are administered systemically and the observed outcome reflects the net effect of all the metabolic, cellular, and molecular changes induced by the treatment.

### Continuous subcutaneous infusion of irinotecan or mitoxantrone for 14 days

#### Filling and implanting of osmotic minipumps

On Day 5, i.e., 1 day after the end of the i.p. VEGF treatment, osmotic minipumps (Model 2ML2, with constant pumping rate of 5.0 μL/h for 14–15 days; Alzet® Osmotic Pumps, Mountain View, CA, USA) were filled under sterile conditions with the irinotecan or mitoxantrone solution or the appropriate vehicle. After storage in sterile 0.9% (w/v) NaCl overnight at 37 °C, the pumps were surgically implanted s.c. on the backs of rats that had been anesthetized with inhaled isoflurane (Forene®; Abbott, Abbott Park, IL, USA). The skin incision made for pump implantation was sutured immediately post-implantation using silk thread. As the animals gained weight physiologically during the experimental period, the actual dosage of drug per kg of body weight was higher than the average dosage at the beginning of the infusion period and below the average dosage at the end of the infusion period. Continuous infusion, as used in the present study, can be viewed as an extended form of metronomic treatment.

Irinotecan (Campto®; Pfizer, New York, NY, USA), which was placed in a tube that was wrapped in aluminum foil after dilution, was administered at 50 mg/kg/wk in the vehicle, which consisted of NaCl 0.9% (w/v), sorbitol 0.45% (w/v), and lactic acid 0.09% (w/v) (pH 3.5). Rats that received vehicle alone (controls) were included in each experiment.

Mitoxantrone (Novantrone®; Pfizer) was administered at 0.5 mg/kg/wk in the vehicle, which consisted of NaCl 0.8% (w/v), Na-acetate 0.005% (w/v), and acetic acid 0.046% (w/v) (pH 3.0–4.5), with or without N-acetylcysteine (NAC; Sigma Chemical Co., St. Louis, MO, USA) at 192 mg/kg/day. NAC, which is a potent anti-oxidant, has been widely used as a tool for investigating the roles of reactive oxygen species (ROS) in biologic and pathologic processes. NAC was administered to two of the four groups of animals (Table 2) without affecting body-weight gain (data not shown). Rats that received vehicle alone or vehicle plus NAC (controls) were included in each experiment.

The dosage of a drug per body surface area (mg/m^2^) may be useful for comparing drug toxicities between species, e.g., between laboratory animals and humans (24). For a rat that weighs 250 g, the dosage in mg/kg multiplied by seven yields the approximate dosage in mg/m^2^. Thus, the dosages used for each 250-g animal in the angiogenesis experiment correspond to ∼350 mg/m^2^ per week for irinotecan and ∼3.5 mg/m^2^ per week for mitoxantrone in a human.

### Angiogenesis quantification

After 14 days of continuous drug infusion, four membranous, virtually transparent (window-like) samples from the most distal part of the small-gut mesentery directly proximal to the ileo-cecal valve, were collected and examined microscopically after being spread intact on objective slides and stained immunohistochemically (23). Normally, this tissue has a thickness of only 5–10 μm in its avascular parts, making the whole microvessel network virtually two-dimensional. The surrounding fatty tissue distinctly delineates each window. The entire vasculature of each of the four mesenteric windows per animal was visualized using a primary monoclonal antibody against rat endothelium, MRC OX-43 (19), which labels the vascular endothelium in all tissues of the rat, except that of the brain capillaries. This procedure allows the reliable identification of even the smallest microvessels.

For the analysis of objective unbiased microvessel variables, microscopic morphometry and computerized image analysis were employed in a blinded fashion. First, the total area of each sampled mesenteric windows was measured. The following variables were then measured in each window (23): (i) the percentage vascularized area (VA), which is a measurement of the spatial extension of the network; and (ii) the microvascular length (MVL), which is a composite measurement of microvessel density. The total microvascular length (TMVL) was calculated according to the following formula: TMVL = VA × the mean MVL per treatment group.

### Statistical analysis

The non-parametric Mann–Whitney *U*-test was used to analyze unpaired (two-tailed) observations. A mean of four mesenteric windows per animal was used as the independent data-point for each variable of the mesenteric window. The criterion for statistical significance was p ≤ 0.05.

## Results

### Effect of 14-day continuous irinotecan or mitoxantrone infusion on angiogenesis

The animals behaved normally in both experiments. The doses used did not produce signs of toxicity (see below). Chemotherapy did not affect the area (size) of the individual sampled mesenteric windows, as compared with those of the vehicle controls (data not shown). Therefore, direct comparisons with the vehicle controls can be made for all the angiogenesis variables presented.

Irinotecan infusion increased the value for VA (to 168% of the value for the vehicle control; p ≤ 0.05), MVL (to 121% of the value for the vehicle control; p > 0.05), and TMVL (204% of the vehicle control; p ≤ 0.005) (Table 1).

**Table 1:** Effects of 14 days of subcutaneous infusion of irinotecan (50 mg/kg/week) on VEGF-mediated angiogenesis in the rat

	Vehicle control	Irinotecan
		
	% of corresponding value for vehicle control	
VA	13.71 ± 2.00	23.06 ± 4.77^1^
	100	168
MVL	0.89 ± 0.09	1.08 ± 0.08
	100	121
TMVL	12.20 ± 1.44	24.90 ± 3.74^2^
	100	204

Vehicle used: NaCl 0.9% w/v, sorbitol 0.45% w/v, lactic acid 0.09% w/v (pH 3.5).

MVL, microvascular length, a composite measure of microvessel density; TMVL, total microvascular length (VA x MVL); VA, vascularized area, a measure of microvessel spatial extension.

1 p ≤ 0.05,

2 p ≤ 0.005 compared with the vehicle control. There were 14 animals in each group. Data shown as mean ± SEM.

At sacrifice, the mean body weight of the irinotecan-treated animals was 1% lower than that of the vehicle controls (381.2 ± 8.2 g, mean ± SEM), which during the 14 days of s.c. infusion treatment showed a mean increase in weight of 60.6 g/week (the difference in mean body weight at any time-point during the s.c. infusion was ≤1% between the treatment groups).

Mitoxantrone infusion without NAC significantly increased the TMVL, as compared with the appropriate vehicle control (to 169% of the TMVL for the vehicle control; p ≤ 0.03) (Table 2). In animals that were not exposed to mitoxantrone, infusion of the vehicle plus NAC led to increases in the VA (141% of the vehicle control; p > 0.05), MVL (144% of the vehicle control; p > 0.05), and TMVL (203% of the vehicle control; p ≤ 0.025) (Table 2).

**Table 2:** Effects of 14 days of subcutaneous infusion of mitoxantrone (0.5 mg/kg/week) on VEGF-mediated angiogenesis

	Group 1	Group 2	Group 3	Group 4
	
	Vehicle without NAC n = 10	Vehicle with NAC n = 8	Mitoxantrone without NAC n = 12	Mitoxantrone with NAC n = 12
	
	% of corresponding value for Group 1			
VA	14.98 ± 2.13	21.08 ± 4.64	19.07 ± 2.98	16.97 ± 3.93
	100	141	127	113
MVL	0.79 ± 0.12	1.14 ± 0.14	1.05 ± 0.09	0.94 ± 0.07
	100	144	133	119
TMVL	11.83 ± 1.68	24.03 ± 5.29^1^	20.02 ± 3.13^2^	15.95 ± 3.69
	100	203	169	135

Vehicle used: NaCl 0.8% w/v, Na-acetate 0.005% w/v, acetic acid 0.046% w/v (pH 3.0–4.5). NAC, N-acetylcysteine.

1 p ≤ 0.025;

2 p ≤ 0.03 compared with Group 1. Data are shown as mean ± SEM.

At sacrifice, the mean body weight of the mitoxantrone-treated animals was 1% lower than that of the vehicle controls (339.1 ± 6.6 g, mean ± SEM) (see Group 1 in Table 2), which over the 14 days of s.c. infusion showed a mean increase in weight of 62.1 g/week (the difference in mean body weight among the four treatment groups at any time-point during the s.c. infusion was ≤2%).

## Discussion

### Important aspects of the assay used

As the inhibition of angiogenesis limits tumor growth and *vice versa* (4, 25), it can be argued that a study of the angiogenesis-modulating effects *per se* of any potential anti-tumor drug should be conducted in a tumor-free tissue. VEGF is a key angiogenic factor in tumor development and appears to be a mediator of angiogenic pathways that involve a number of other pro-angiogenic factors. As discussed below, the rat mesentery used in the present study appears to be a satisfactory surrogate model for tumor angiogenesis, in part because the ECs involved in tumor angiogenesis use the same signaling pathways as the ECs that are involved in non-tumor angiogenesis (26).

The assay used compares well with other mammalian *in vivo* angiogenesis assays in regard particularly to important features such as the adult test tissue is natively vascularized and lacks significant physiologic angiogenesis; minimal trauma, if any, is inflicted upon the tissue; truly quantitative variables are measured, which allows sound statistical analysis regarding dose-response and molecular-activity studies; sprouting angiogenesis occurs, which is the predominant form in tumors; toxic data are easily accumulated as rats grow robustly physiologically in adulthood; the assay is highly suited to studies of the combined effects on angiogenesis of agents that are administered systemically in a concurrent or sequential fashion; and the assay replicates the clinical situation, as the angiogenesis-modulating test drugs are administered systemically and the responses observed reflect the net effect of all the metabolic, cellular, and molecular alterations induced by the treatment. The assay is discussed in invited review articles in terms such as of biological relevance, ethical aspects, economy and labor intensity (17, 18), and is demonstrated in a video presentation with attached detailed protocol (27). However, this assay does not take into account all aspects of tumor-induced angiogenesis, e.g., the presence of multiple pro-angiogenic factors, which are usually present in advanced cancers, the chaotic microvessel patterns and perfusion of the tumor vasculature, the cellular composition of the perivascular stroma, and the heterogeneity of the ECs within individual tumors. Significant differences in pO_2_ and pH are also noted between tumorous and non-tumorous tissues. Nevertheless, in a rat model of syngeneic prostate cancer, the present assay for VEGF-mediated angiogenesis revealed a close correlation between the anti-angiogenic effects in the tumor-free mesentery and the anti-tumor effects resulting from continuous s.c. infusion of paclitaxel (28). Moreover, mouse ascites tumor cells induce a repertoire of biologic responses in the mesentery vasculature, which can be attributed entirely to VEGF (29). Additional confirmation that inhibition of VEGF-induced angiogenesis in the tumor-free mesentery reflects significant events leading to the inhibition of tumor growth comes from the fact that systemic treatment with bovine iron-unsaturated lactoferrin suppresses VEGF-induced angiogenesis in the rat mesentery model (30), as well as cancer cell-induced angiogenesis in a mouse dorsal air sac assay (31).

### How to study anti-angiogenesis in tumors?

The alleged target of low-dosage metronomic chemotherapy is the angiogenically activated normal ECs. Whether or not the ECs in tumors and in normal tissues are genetically and functionally identical remains controversial (32), even though the tumor endothelium exhibits a phenotype of angiogenically activated ECs in normal tissue, as reflected in the high levels of expression of angiogenic molecules, such as VEGFR, the angiopoietin receptor Tie2, and the adhesive molecules ICAM-1, E-selectin, and CD44 (26). It is noteworthy that tumor angiogenesis is not a well-defined concept, as in tumors, there is a constant cross-talk between the genetically heterogeneous neoplastic cells, the ECs, stromal cells (including fibroblasts), leukocytes (including lymphocytes), sentinel immune cells (mast cells, macrophages and dendritic cells), platelets, and the extracellular matrix. Mainly due to the dynamic genetic heterogeneity among the neoplastic cells, angiogenesis-related features may be tumor-specific in principle. It should also be remembered that there may be substantial differences in angiogenesis between authochthonous tumors (originating in the place found), which represent the main tumor type seen in the clinic, and transplanted tumors (also including xenograft tumors) (33). Moreover, many of the tumor models used in pre-clinical studies are clearly dissimilar in important respects to most human tumors. There are also distinct differences between inbred mouse strains in terms of responsiveness to growth factor-stimulated angiogenesis (34). These factors make comparisons between studies using different tumor models difficult. From the clinical perspective of anti-angiogenic therapy, it would appear that the predictive value for translation into the clinic of data obtained in a number of transplanted tumor models is limited.

### Divergent angiogenesis-modulating effects of irinotecan and mitoxantrone

We report that low-dosage continuous infusion of either irinotecan or mitoxantrone enhances VEGF-induced angiogenesis in the normal rat mesentery. This is in contrast with previous reports of both mitoxantrone and irinotecan having anti-angiogenic activities in a variety of *in vivo* and *in vitro* models. Mitoxantrone, for example, has been reported to suppress angiogenesis in the tumor-free surgical rat cornea pocket and in the embryonic chick chorioallantoic membrane assays *in vivo* (35, 36). Irinotecan has been found to inhibit angiogenesis in several *in vivo* tumor xenograft models in mice (6, 32, 37–39), and in EC cultures (6, 40). In some of these studies, low-dosage irinotecan treatment inhibited hypoxia-inducible factor (HIF)-1 alpha and/or up-regulated the anti-angiogenic protein thrombospondin-1. We speculate that the current conflicting data hinge on the fact that the rat mesentery assay encompasses a unique set of features among *in vivo* angiogenesis models (such as an adult natively vascularized visceral tumor-free tissue that is influenced minimally by inflammation and not exposed to either surgery/trauma or high levels of oxygen). Furthermore, the assay allows objective, unbiased variables to be assessed quantitatively. The rat mesentery model compares favorably with most of the assays used in the cited articles (17, 18). However, the relevance of the various pre-clinical assays to human pathobiology remains to be established.

There are numerous discrepancies in the literature concerning the angiogenesis-modulating effects of various standard cytotoxic agents in tumors and tumor-free assays (19, 41, 42). This is not surprising considering the differences in species used, experimental design, test tissues used, modes of administration, dosages, and the biological features of the assays (17). In many of these models used for the study of the effects of chemotherapeutics on angiogenesis, trauma is induced, which affects the test tissue. This is a problem because cell injury generates ROS, which fragment the extracellular matrix component hyaluronan, which in turn synergizes with ROS to activate the innate immune system and further promote ROS production and angiogenesis (43). Assays in which the new blood vessels are close to tissue-air interfaces may, moreover, allow exposure to artificially high concentrations of oxygen, with consequential ROS production, as in the corneal micropocket and chick embryonic chorioallantoic membrane assays (17). Major models of angiogenesis occasionally yield dissimilar, and in some cases even contradictory outcomes (18).

### Reactive oxygen species, anti-oxidants, chemotherapy and angiogenesis *in vivo*

Transcription factors and genes involved in angiogenesis are regulated by ROS and the signaling properties of ROS are due, in part, to reversible oxidative inactivation of redox-sensitive target proteins, such as nuclear factor kappa B (NF-kB) (44, 45). Normally, there is a balance between the production and removal of ROS. An imbalance in favor of the pro-oxidative state is often referred to as ‘oxidative stress’. In tumors, ROS, such as superoxide anion and hydrogen peroxide (H_2_O_2_), are found in large quantities (46). The drugs of many classes of chemotherapeutics generate in a dose-dependent manner a high level of oxidative stress in biological systems (47). Importantly, ROS appear to have biphasic effects on angiogenesis, in that high levels induce cell death, apoptosis, and senescence of ECs, while low levels function as intracellular signaling molecules in ECs.

These ROS-dependent intracellular signaling molecules play an important role both in physiologic as well as pathologic angiogenesis by mediating cell growth, migration, and differentiation, as well as the gene expression involved in angiogenesis (44, 48). In fact, H_2_O_2_ potently induces VEGF expression in wounds; this oxidant-induced VEGF expression is independent of HIF-1 alpha (49). Low levels of H_2_O_2_ also play a role in the angiogenic switch (48) and are essential for the trophic effects of a wide variety of cytokines (50).

Studying the effects of chemotherapy on angiogenesis is complex because (i) VEGF stimulation *per se* increases ROS production via the activation of Rac1-dependent NADPH-oxidase in ECs (44). Moreover, ROS are important for the activation of VEGF-induced cSrc (a master cellular control protein that is involved in cell proliferation, differentiation, motility, angiogenesis, and other functions) activation, phosphorylation of VE-cadherin, and protein kinase B/Akt in ECs, thereby additionally stimulating angiogenesis (44). ROS at low levels generated by H_2_O_2_ under normoxia induce HIF-1 alpha transcription via binding of NF-kB (45); (ii) ROS are able to instigate angiogenesis (44, 48). As demonstrated in the present study, NAC significantly enhanced VEGF-mediated angiogenesis in animals that were not exposed to mitoxantrone (Table 2), which suggests that redox-related effects that affect VEGF-induced angiogenesis operate in this model. Although the mechanism underlying this apparently pro-angiogenic effect of NAC remains to be elucidated, it may be related to reducing the oxidative stress induced by VEGF to a more optimal angiogenesis-stimulating level.

### Reactive oxygen species, anti-oxidants, and angiogenesis *in vitro*

Studies of ROS-associated processes in cultured ECs are not uncomplicated given that: (i) cell culturing influences antigen expression in human ECs, which means that the phenotype changes early in response to culture conditions (51); (ii) the media used to grow ECs contain growth factors, i.e., stimulatory agents that may influence the cell phenotype (51); (iii) oxidative stress occurs in all cell cultures; in particular, riboflavin, which is a necessary component of cell culture media, is responsible for the generation of ROS (52); and (iv) there are major differences in characteristics of ECs derived from different tissues, making comparisons between experiments difficult (53, 54). Considering the intricate relationships between the production of ROS by chemotherapeutics, the spontaneous production of ROS in cell cultures and the phenotypic modification and diversity of cultured ECs, the data obtained from *in vitro* experiments on the alleged direct effects of chemotherapeutics on ECs (often referred to as ‘*in vitro* angiogenesis’) should probably be interpreted with caution.

### Metronomic chemotherapy exerts diverse anti-tumor effects

Several potentially independent modes of anti-angiogenic/anti-tumor effects may operate individually or in combination during low-dosage continuous chemotherapy. These effects include: (i) angiogenesis modulation via direct effects of the cytotoxic agent on the vascular ECs, circulating ECs, and EC precursors in the bone marrow; (ii) direct effects of the cytotoxic agent on neoplastic and tumor stromal cells, with consequent influence on tumor angiogenesis; (iii) effects on platelets, which play an important role in angiogenesis in that they accumulate angiogenesis-regulating proteins in two sets of alpha-granules, with positive regulators in one set and negative regulators in the other set (55); these regulators can be released separately, depending on the type of therapeutic molecule used; (iv) selective depletion of specific immune effector cells with the potential to evoke tumor immunity (56); (v) effects on the redox-state, which may be crucial for the outcome of angiogenesis modulation following low-dosage treatment with certain cytotoxic agents (19); and (vi) the induction of the potent endogenous anti-angiogenic proteins endostatin and thrombospondin in ECs and possibly in other cells (16, 57, 58).

Clearly, analyses of normal tissues and tumors are needed to clarify the relative contributions of these modes and their respective effects on the anti-tumor outcome of continuous chemotherapy. Elucidation of the influences of these diverse anti-tumor modes would facilitate the design of effective therapeutic strategies.

## Conclusions

In the literature, it is often stated that metronomic chemotherapy has anti-angiogenic effects. Investigating the effects of chemotherapeutics on angiogenesis *per se* is, however, complicated because: (i) in addition to affecting cellular metabolism, cytotoxic agents can up-regulate and down-regulate genes and generate ROS in targeted ECs (the production of ROS is dose-dependent and possibly tissue-dependent); and (ii) *in vitro*, spontaneous generation of ROS in cells grown in culture media has an impact on the results. The effect on VEGF-mediated angiogenesis of metronomic mono-chemotherapy in the rat mesentery model is drug-specific (and dose-related). Thus, previous studies have reported that these agents are pro-angiogenic, e.g., ironotecan and, mitoxantrone (as reported here), as well as cisplatin and 5-fluorouracil (19, 42), anti-angiogenic, e.g., cyclophosphamide, paclitaxel, and vinblastine (19, 41), or have no effect on angiogenesis, e.g., doxorubicin and epirubicin (19, 20). Low-level oxidative stress in ECs appears to induce pro-angiogenic effects following certain types of low-dosage metronomic chemotherapy in this model. Whether or not these findings hold true for tumor angiogenesis remains to be clarified.

The present data suggest that when low-dosage, metronomic, single-drug chemotherapy with irinotecan or mitoxantrone produces an anti-tumor outcome one has to take into account that several separate modes of anti-tumor effects, including those unrelated to angiogenesis modulation, may operate in parallel.

## Notes

Financial support for this study was provided by the Swedish Cancer Foundation (grants 02 0503 and 06 0007 to PA) and the King Gustav V Jubilee Clinic Cancer Research Foundation. The authors declare that they have no conflicts of interest.

## Abbreviations

bFGF: basic fibroblast growth factor, FGF-2
EC: vascular endothelial cell
HIF: hypoxia-inducible factor
i.p.: intraperitoneal
MVL: microvascular length
NAC: N-acetylcysteine
NF-kB: Nuclear factor kappa
ROS: reactive oxygen species
s.c.: subcutaneous
TMVL: total microvascular length
VA: vascularized area
VEGF: vascular endothelial growth factor-A, isoforms 164 and 165.
